# Commentary: Episodic Future Thinking about the Ideal Self Induces Lower Discounting, Leading to a Decreased Tendency toward Cheating

**DOI:** 10.3389/fpsyg.2020.01027

**Published:** 2020-05-19

**Authors:** Manuela Sellitto

**Affiliations:** Comparative Psychology, Institute of Experimental Psychology, Heinrich Heine University Düsseldorf, Düsseldorf, Germany

**Keywords:** morality, imagination, intertemporal choice, cheating, delinquency, ideal self, episodic future thinking, delay discounting

In an interesting study, Wu et al. (2017) explored a novel facet of the effect of episodic future thinking (EFT) over delay discounting (DD) (Peters and Büchel, 2010; Benoit et al., 2011). They specifically tested the idea that thinking about the ideal self would facilitate the consideration of future consequences, and, in turn, would reduce DD and so the likelihood of making delinquent choices. Indeed, as Wu et al. (2017) propose, delinquency could be considered in itself as an intertemporal choice between the immediate gains of delinquent behavior and the later costs potentially associated with it (e.g., bad reputation). Thinking about the ideal self was expected to have a larger effect than both general EFT and semantic future thinking as, based on the literature, the authors assumed it to better support goal attainment (see also Donnell et al., 2017). Across two experiments, participants in the EFT groups had to think about aspects of their ideal selves (i.e., physical, social, moral, and psychological) and to pre-experience, vividly, imagined future life events that would occur if the desirable aspects of their ideal selves were realized. Conversely, participants in several control conditions had/had not to generate representations of an ideal self without engaging in mental simulation, or to re-experience present events, or to think about someone else's autobiographical details. The likelihood of engaging in delinquent acts was subsequently measured via both hypothetical scenarios and real opportunities of cheating. Overall, the results showed that imagining and pre-experiencing life events that would be experienced by the ideal self projected into the future reduced significantly the tendency to steeply discount future rewards. This, in turn, mediated a reduced willingness to engage in delinquent activities and cheating tendencies (Wu et al., 2017), suggesting that the ability to delay gratification, giving up current temptations, might have an overarching effect.

Wu et al. (2017) also remark that some questions were left unanswered. For instance, whether thinking about the ideal self is necessary to produce such effect or, instead, the engagement in EFT would be beneficial *per se*, or whether the effect of EFT on DD is temporally invariant. Recently, reduced DD could be observed not only in participants who had imagined future events, but also in those who had remembered past events or imagined, vividly, present events alternative to the current experience, as compared to merely describing the current situation, before making intertemporal choices (Ciaramelli et al., 2019). Although not directed at exploring delinquent behavior, these findings suggest that shifting perspective from the perceptual present toward mentally constructed events can favor larger future outcomes, regardless of the temporal location of the imagined experience. Therefore, if by detaching from the experienced sensorial information via the imagination of an alternative present one could reduce the gap between future events and the current experience, then, based on Wu et al. (2017) findings, this is likely to work even more powerfully via the simulation of an ideal self. It remains, however, still unanswered whether episodic simulation might make a future self/outcome/reward more tangible or rather a present gain less relevant [see also (Parthasarathi et al., 2017)].

It is well-established that EFT and DD rely on a common neural network that encompasses fronto-medial regions, and lesional studies have clearly shown that disruption of such areas results in both increased DD (Frost and Mcnaughton, 2017) and poor EFT (McCormick et al., 2017). More importantly, structural and functional abnormalities in these regions and their associated striatal connectivity have been found in pathological populations characterized by psychopathy and delinquent behavior (Hosking et al., 2017; Korponay et al., 2017). Wu et al. (2017) findings, although only behavioral, well fit into this neural background, shading additional lights on a possible psychological mechanism underlying antisocial behavior. Specifically, they propose the idea that the degree of sensitivity to immediate outcomes, identifiable as individual trait and impulsivity facet, might drive socially relevant problems, from unhealthy behavior (e.g., overeating, gambling, drug addiction) to antisocial conduct disorders (Odum, 2011; Jimura et al., 2013). On a broader view, a key point is that this might have also clinical relevance, as it reveals potentially effective ways of reducing DD and therefore, possibly, delinquent attitude (see also Snider et al., 2016; Bulley and Irish, 2018; Madden, 2018; Scholten et al., 2019).

Wu et al. (2017) showed that simulating the ideal self could reduce the likelihood of making delinquent choices and of cheating: this could be easily implemented in interventions oriented at nudging toward moral and social behavior. Putting (Wu et al., 2017) and other (Donnell et al., 2017; Ciaramelli et al., 2019) findings together, it could be, therefore, that thinking about an alternative ideal self—self-projection—might be sufficient for promoting optimal intertemporal choice, independent of time frame, and thus even encouraging the mere imagination/construction of alternative, present ideal selves could help improving and fostering socially relevant behaviors. As an example, if someone committed cyberbullying, she could be trained in thinking about her ideal self, no matter whether located in the present or in the future time. On this regard, it is worth pointing out that self-projection relies on (positive) autobiographical memory (Lempert et al., 2017). Would, then, such a treatment—imagining/simulating the ideal self—work in special cases like repeated offenders? Here, it might still be possible to simulate alternative—ideally better—current situations, without implicating memories of the self. This could be sufficient to improve the ability of forecasting consequences of one's own actions, thus endorsing more farsighted and morally acceptable decisions (Ciaramelli and Di Pellegrino, 2011).

Finally, it is worth noting that Wu et al. (2017) tested Eastern participants. Considering the differences in DD across different cultural backgrounds (Takahashi et al., 2009), it might also be that the elicitation/framing of an ideal self—in the moral and social way—has a different impact in Eastern and Western societies and their associated criminal behavior incidence. It would thus be crucial to see future studies addressing this issue.

## Author Contributions

The author confirms being the sole contributor of this work and has approved it for publication.

## Conflict of Interest

The author declares that the research was conducted in the absence of any commercial or financial relationships that could be construed as a potential conflict of interest.
